# In Vitro Phytobiological Investigation of Bioactive Secondary Metabolites from the *Malus domestica*-Derived Endophytic Fungus *Aspergillus tubingensis* Strain AN103

**DOI:** 10.3390/molecules27123762

**Published:** 2022-06-11

**Authors:** Hassan Mohamed, Weaam Ebrahim, Mona El-Neketi, Mohamed F. Awad, Huaiyuan Zhang, Yao Zhang, Yuanda Song

**Affiliations:** 1 Colin Ratledge Center for Microbial Lipids, School of Agricultural Engineering and Food Science, Shandong University of Technology, Zibo 255000, China; zhyuan004@126.com (H.Z.); zhangyao@sdut.edu.cn (Y.Z.); 2 Department of Botany and Microbiology, Faculty of Science, Al-Azhar University, Assiut 71524, Egypt; m.fadl@tu.edu.sa; 3 Department of Pharmacognosy, Faculty of Pharmacy, Mansoura University, Mansoura 35516, Egypt; weaamnabil@mans.edu.eg (W.E.); melneketi@mans.edu.eg (M.E.-N.); 4 Department of Biology, College of Science, Taif University, Taif 21944, Saudi Arabia

**Keywords:** *Aspergillus tubingensis*, 18S rRNA, secondary metabolites, antimicrobial, cytotoxicity

## Abstract

Endophytic fungi including black aspergilli have the potential to synthesize multiple bioactive secondary metabolites. Therefore, the search for active metabolites from endophytic fungi against pathogenic microbes has become a necessity for alternative and promising strategies. In this study, 25 endophytic fungal isolates associated with *Malus* *domestica* were isolated, grown, and fermented on a solid rice medium. Subsequently, their ethyl acetate crude extracts were pretested for biological activity. One endophytic fungal isolate demonstrated the highest activity and was chosen for further investigation. Based on its phenotypic, ITS ribosomal gene sequences, and phylogenetic characterization, this isolate was identified as *Aspergillus tubingensis* strain AN103 with the accession number (KR184138). Chemical investigations of its fermented cultures yielded four compounds: Pyranonigrin A (1), Fonsecin (2), TMC 256 A1 (3), and Asperazine (4). Furthermore, ^1^H-NMR, HPLC, and LC-MS were performed for the identification and structure elucidation of these metabolites. The isolated pure compounds showed moderate-to-potent antibacterial activities against *Pseudomonas aeruginosa* and *Escherichia* *coli* (MIC value ranged from 31 and 121 to 14.5 and 58.3 μg/mL), respectively; in addition, the time–kill kinetics for the highly sensitive bacteria against isolated compounds was also investigated. The antifungal activity results show that (3) and (4) had the maximum effect against *Fusarium solani* and *A. niger* with inhibition zones of 16.40 ± 0.55 and 16.20 ± 0.20 mm, respectively, and (2) had the best effect against *Candida albicans*, with an inhibition zone of 17.8 ± 1.35 mm. Moreover, in a cytotoxicity assay against mouse lymphoma cell line L5178Y, (4) exhibited moderate cytotoxicity (49% inhibition), whereas (1–3) reported weak cytotoxicity (15, 26, and 19% inhibition), respectively. Our results reveal that these compounds might be useful to develop potential cytotoxic and antimicrobial drugs and an alternative source for various medical and pharmaceutical fields.

## 1. Introduction

Apple trees are among the most important fruit trees and are widely grown and cultivated throughout the temperate regions, which ultimately makes apples the most produced temperate fruit from the northern to southern hemispheres [1]. Apple has been reported in the tropical and subtropical climates [2]. During the life cycle of plants, at some point endophytic fungi gain entry to their tissues, but these microbes do not cause any damage to them [3,4]. These microbes are important for balancing the ecosystem and benefit the plants by increasing their growth rate [5]. Endophytic fungi also represent a promising and still largely untapped reservoir of biologically active secondary metabolites with potential for exploitation and application in the pharmaceutical industry [6].

*Aspergillus* spp. are considered some of the oldest fungal members known to science, and are widespread in different habitats (plants, marine, air, dust, and soil), and have the ability to survive under extreme natural environmental conditions [7]. Black aspergilli are important due to their fast growth, high abundance, pH tolerance [8], and production of toxic metabolites, especially napthopyrones, ochratoxin, and malformins [9]. *A. tubingensis* and *A. niger* are presumably the most well-known black aspergilli. Additionally, they are widely used in many studies to explore their secondary metabolites and due to their common name black *Aspergillus*, which has led to their consequent and incorrect name as *A. niger*. In addition, *A. tubingensis* represents a good alternative candidate for metabolite production in industrial fermentation and is already used for some applications, such as bioactive molecules, enzyme production, and specialized metabolites such as asperazine, which are considered as diagnostic chemical markers [10,11]. Due to their highly similar morphological features and inadequate molecular identification, the classification of these species has become more difficult, leading to misclassifications and differing phylogenetic trees [8]. The classification-based taxonomy of black aspergilli can be successfully employed in accordance with a mixture of phenotypic and chromatographic approaches and chemotaxonomic, molecular, and metabolite profiling [12]. Therefore, new molecular DNA-based techniques for accurate and rapid identification have been studied and applied in recent decades [12]. The wild distribution of black aspergilli makes it grow and damage crops and foods worldwide, including peanuts, raisins, corn, onions, apples, mango, and even dried meat products [13]. The screening programs for food and feed were launched after the recent discovery that black aspergilli have the potential to produce toxic substances [14,15]. Furthermore, secondary metabolites of the naptho-γ-pyrones (NγPs) family are the most abundant in this group [11,16]. In the various systems, the biological effects of several NγPs have been investigated [17], and they were reported to have cytotoxic, antibacterial, antifungal, and antitumoral activities [18,19].

Multiple (NγPs), including aurasperone A, asperpyrone E, fonsecin, dianhydroaurasperone C, fonsecinone A, ustilaginoidin A, and fonsecin B, were isolated from a non-toxigenic *A. tubingensis* strain G131 [20]. In another study, dimeric NγPs, named rubasperone A, B, and C, together with rubrofusarin and rubrofusarin B, were isolated from the mangrove endophytic fungus *A. tubingensis* strain GX1-5E [16]. Moreover, asperazine is another NγPs; it is an uncommon asymmetrical diketopiperazine dimer isolated from *A. tubingensis*, which was proposed as a special chemical marker to distinguish *A. tubingensis* from *A. niger*. In addition, the genomic analyses indicated that *A. tubingensis* has the capability to produce asperazine, while none of the *A. niger* strains have this capacity [21].

The purpose of the current study was isolation, structure elucidation, and characterization of active secondary metabolites from the endophytic fungus *A. tubingensis* strain (AN103) associated with *M. domestica* from Southern Russia. Furthermore, antimicrobial, time–kill kinetics, and cytotoxic activities of the obtained pure compounds were also investigated.

## 2. Results

### 2.1. Isolation of Endophytic Fungi and Preliminary Screening for Bioactivity

A total of 25 fungal strains associated with stem parts (40 fragments) of fully mature *M. domestica* trees were isolated at different locations in the Saratov region. Those isolates were identified based on the phenotypic and morphological combination of different protocols, and represented four genera, including: *Alternaria* spp., predominantly with eight isolates (32%), followed by *Aspergillus* spp., with six isolates (24%), *Penicillium* spp., with our isolates (16%), *Fusarium* spp., with four isolates (16%), and *Cladosporium* spp., with three isolates (12%). These isolated fungi were fermented in a solid rice medium and extracted with EtOAc solvent. The crude extracts were then evaluated for the antimicrobial activities against some pathogenic bacteria. The results obtained (Table S1) show that the antibacterial activities demonstrated varying degrees of inhibition, and significant differences were observed as compared to the control. The results recorded high antibacterial activity against pathogenic strains that were tested (*E. coli*, *P. aeruginosa*, *Staphylococcus aureus*, and *Bacillus subtilis*), with inhibition zones that ranged between 13.3 ± 0.2 and 29.5 ± 0.8 mm. Other crude extracts of endophytic fungi showed significant inhibition when compared with the control. Most *A. alternata* and *C. cladosporioides* extracts had almost no effect on the growth *E. coli*, while *F. tricinctum* and *A. fumigatus* showed no activity on *P. aeruginosa.* Overall, the crude extract of *A. tubingensis* AN103 showed high broad-spectrum activity among other tested fungal extracts; therefore, this strain was selected for further study.

### 2.2. Morphological Identification of the Most Promising Isolate

**Figure 1:**
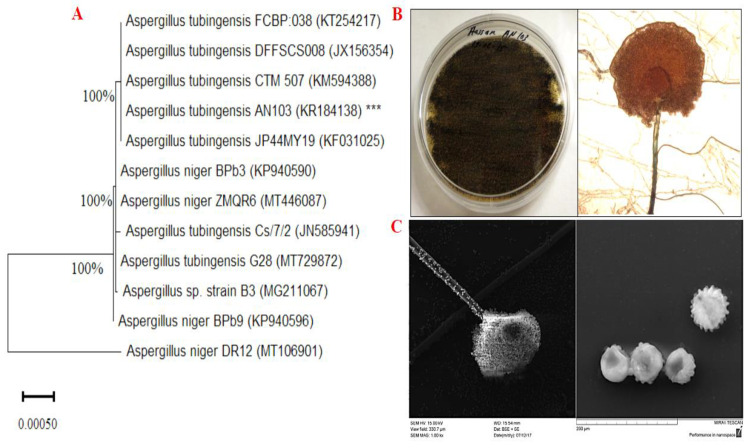
(**A**) The neighbor-joining (NJ) phylogenetic tree based on ITS gene sequences of AN103, with closely related strains accessed from the GenBank using BLASTN tool. These sequences were aligned using ClustalW. Bootstrap values included 500 replicates for the NJ method using MEGA software (version 11.0.6). *** indicates selected strain. (**B**) Morphological features of AN103 strain on agar plates. (**C**) Spore and sporangium of AN103 strain under SEM.

Based on the preliminary biological activities of all isolated endophytic fungi, the most promising strain, AN103, was selected for further confirmation. AN103, morphologically identified as *A. tubingensis*, has a similar morphology to *A. niger*. However, a characteristic of *A. tubingensis* is the production of white-to-pink-colored sclerotia, and it could be classified based on this morphology, but this characteristic is seldom observed in *A. niger* species. In the early culture stages (3–5 d), white mycelia and radial villus were observed on the edges of each purified colony. The characteristics of the mycelium, such as pitchy at the head while white on the reverse side of the PDA plate, were observed after seven days. The round and radial vesicle of AN103 was observed. Hyphal diaphragms were clearly visible with a microscope (Figure 1).

### 2.3. DNA-Based Identification and Phylogenetic Analysis of A. tubingensis AN103

The molecular identification and phylogenetic description of the most promising isolate, *A. tubingensis* AN103, were determined using ITS gene sequencing. The partial 18S rRNA gene sequence of AN103 exhibited excellent homology between phenotypic features and 18S rRNA sequence data. BLAST analysis revealed that the AN103 strain belonged to *Aspergillus* spp., and the sequence was deposited in the NCBI database with the accession number KR184138. The selected strain, AN103, demonstrated the highest 18S rRNA gene sequence similarities (100%) with most *A. tubingensis* strains such as (KT254217, JX156354, KM594388, and *A. tubingensis* KF031025). *A. niger* strain DR12 with the accession number (MT106901) was used as the outgroup. Based on the neighbor-joining method (Figure 1), the morphology and genomic data showed that the AN103 strain represented a strain of the genus *Aspergillus*, which was referred to as *A.* *tubingensis* strain AN103. The identified strain was deposited in the culture collection at Botany and Microbiology Department, Faculty of Science at Al-Azhar University, with the institutional number AU-AN103. AN103 was observed to be elliptical with intercession in the head, with a 45–69 mm diameter of vesicle conidial ornamentation, echinulate and warty, as determined by SEM analysis. Conidia were described as globular with a fine wrinkled texture. Sporangia of AN103 consisted of many spiny spores (Figure 1).

### 2.4. Fermentation and Fractionation of the EtOAc Extract and Its Activity

**Figure 2:**
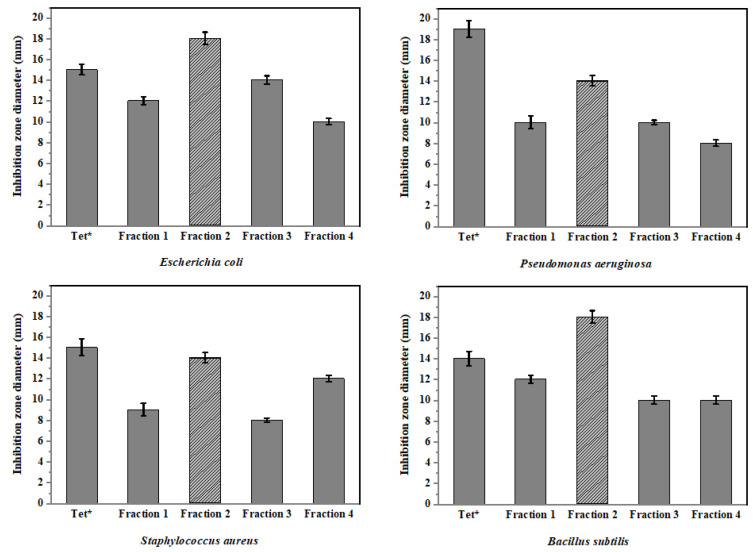
Antibacterial assay by disc diffusion method of the fractions (Figure 1, Figure 2, Figure 3 and Figure 4) from the selected fungal isolate against tested bacteria. Tet* (tetracycline was used as a positive control). Error bars represent standard error of the means (*n* = 3).

**Figure 3:**
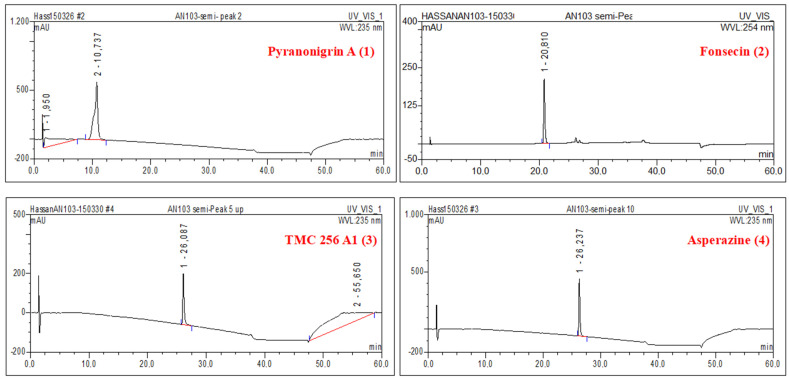
HPLC chromatograms of the isolated pure compounds (1–4), from EtOAc extract active fraction of *A. tubingensis* NA103.

**Figure 4:**
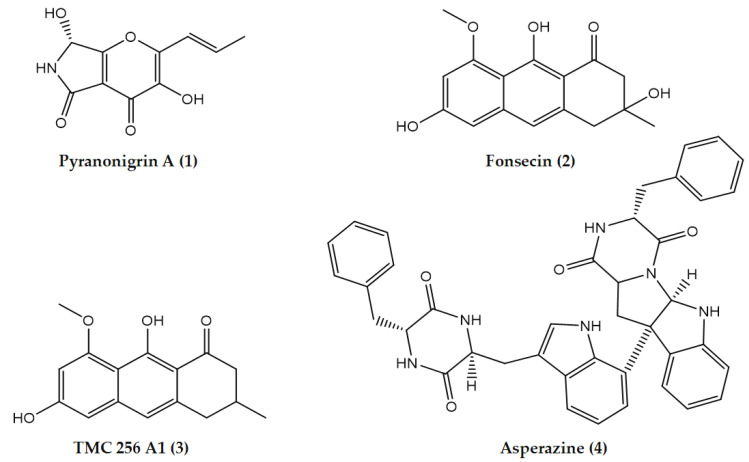
The chemical structures of the isolated pure compounds from the EtOAc extract active fraction of *A. tubingensis* NA103.

The AN103 strain was grown on a solid rice medium (five flasks), and the EtOAc extracts (2.5 g) were separated by silica gel for vacuum liquid chromatography (VLC), followed by partial purification via thin-layer chromatography (TLC) plates, affording four different fractions (Figure 1, Figure 2, Figure 3 and Figure 4). The obtained fractions were assessed for antibacterial activities using the disc diffusion method (50 mg/mL). Results found that fraction no. 2 was the most active fraction; it showed a more significant maximum activity than even the positive control (tetracycline 50 µg/mL) used in this experiment against various pathogenic bacteria that were tested with a zone of inhibition from 14 ± 0.7 to 18 ± 0.9 mm (Figure 2). All tested bacteria were susceptible to the obtained fractions with various degrees of inhibition in comparison to the control. Therefore, fraction no. 2 was chosen as the most promising bioactive extract for further detailed investigation.

### 2.5. Isolation and Identification of Pure Compounds from AN103 Active Fraction

The fungal active fraction (0.8 g) obtained from the EtOAc extract of *A. tubingensis* AN103 was chromatographed by Sephadex LH-20, followed by final purification using semi-preparative HPLC. The four known natural products yielded were pyranonigrin A (**1**), fonsecin (**2**), TMC 256 A1 (**3**), and asperazine (**4**). (Figure 3). All the peaks were detected by a strong signal in UV and observed in the MS chromatograms. The four secondary metabolites were identified mainly by co-chromatography along with other spectroscopic methods.

***Compound 1*** was isolated as a white color, and it showed UV (MeOH) λ_max_: 311, 250, and 209 nm. Positive and negative ESI-MS showed molecular ion peaks at *m*/*z* 224.0 [M + H]^+^ (base peak) and *m*/*z* 222.3 [M − H]^−^ (base peak), respectively, indicating a molecular weight of 223 g/mol. The molecular formula C_10_H_9_NO_5_ (calcd: 223.1822) was obtained from HRESI-MS, and the NMR data suggest that this compound is related to naphtha-γ-pyrone. Structural elucidation of pyranonigrin A was based on results of 1D and 2D NMR spectral analysis, including the following ^1^H NMR data δ: 1.92 (3H, br d, J = 7.0, H-3′), 5.72 (1H, br d, J = 8.8, H-7′), 6.45, (1H, dq, J = 15.8, 8.1, H-2′), 6.75, (1H, br d, J = 15.8, H-1′), 6.87, (1H, d, J = 8.8, OH-7),8.62 (1H, br s, NH), and 9.69 (1H, s, OH-3′).

***Compound 2*** was obtained as a yellow solid, and it showed a UV green fluorescence absorbance maximum at λ_max_ (MeOH) 365 nm. The molecular formula C_15_H_14_O_6_ (calcd: 291.08631) was obtained from HRESI-MS. The structure of the isolated compound was elucidated as fonsecin by analysis of NMR and MS data and comparison with the fonsecin. Structural elucidation of fonsecin was based on results of 1D and 2D NMR spectral analysis, which included ^1^H NMR (300 MHz, CDCl_3_), EI-MS *m*/*z* (%) = 290.2 ([M]^+^, 24), 272.2 ([M − H_2_O]^+^, 16), 243.2 (8), 232.1 (21), 189.1 (7), 175.1 (16), 101.1 (15), 85.1 (22), 59.1 (36), 43.1 (100); (+)-ESI MS *m*/*z* (%) = 291 ([M + H]^+^); (−)-ESI-MS *m*/*z* (%) = 289 ([M − H]^−^); and (+)-HRESI-MS *m*/*z* 291.08631 ([M + H]^+^.

***Compound 3*** was isolated as a yellow powder. Rf = 0.47 (CH_2_Cl_2_/5% MeOH); positive and negative ESI-MS showed molecular ion peaks at *m*/*z* 273.1 [M + H]^+^ (base peak) and *m*/*z* 271.4 [M − H]^−^ (base peak), respectively, indicating a molecular weight of 272 g/mol. The molecular formula C_15_H_12_O_5_ (calcd: 272.253) was obtained from HRESI-MS, and the UV and NMR data suggest that TMC 256 A1 is a naphtha-γ-pyrone. Structural elucidation of TMC 256 A1 was based on results of 1D and 2D NMR spectral analysis, which included ^1^H NMR (300 MHz, DMSO-*d*6) EI MS *m*/*z* (%) = 273 [M + H]^+^; 271 [M − H]^−^; UV λ_max_ nm (log *ɛ*) in MeOH: 224 (4.43), 253 (4.43), 276 (4.61), 326 (3.40), and 405 (3.81).

***Compound 4*** was isolated as a white powder, and it showed UV (MeOH) λ_max_ 300, 285, and 255 nm. Positive and negative ESI-MS showed molecular ion peaks at *m*/*z* 665.2 [M + H]^+^ (base peak) and *m*/*z* 663.4 [M − H]^−^ (base peak), respectively, indicating a molecular weight of 664 g/mol. The molecular formula C_40_H_36_N_6_O_4_ (calcd: 664.752) was obtained from HRESI-MS, and the NMR data show that 6.65 d (3.2), 5.84 d (3.2), 6.75 d (7.2), 6.60 ddd (7.6; 7.2; 0.9), 7.10 ddd (7.9; 7.6; 0.9), 6.75 d (7.9), 3.16 dd (10.4; 7.1), A 3.08 dd (13.6; 7.1) B 2.24 dd (13.6; 10.4), 7.96 d (0.9), 3.42 b, 3.02 dd (13.2; 6.8) 2.88 dd (13.2; 5.0), 6.92 d (6.7), 6.97 dd (7.9; 6.7), 7.52 d (7.9), 9.63 d (1.9), 6.96 d (2.5), 3.14 dd (17.3; 4.1) 2.89 dd (17.3; 4.2), 3.38 b, 8.00 d (1.3), 4.02 ddd, (6.9; 4.7; 4.1), 2.98 dd (13.5; 6.9), and 2.70 dd (13.5; 4.7), 7.19 m. The isolated compound was confirmed as asperazine. The isolated pure compounds are illustrated in (Figure 4); in addition, its LC-UV-MS chromatograms and ^1^H NMR spectra are shown in (Figures S1–S8).

### 2.6. Evaluation of Biological Activities of Individual Compounds

#### 2.6.1. Antibacterial Activity

**Figure 5:**
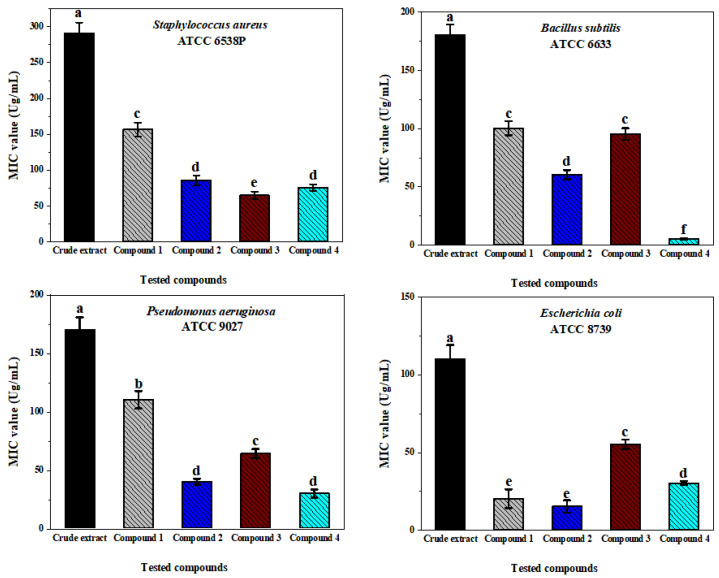
The minimal inhibitory concentration (MIC) of fungal crude extract and its pure compounds against the tested bacterial strains.

The crude extract and its isolated pure compounds obtained from the active fraction (no. 2) of *A. tubingensis* NA103 were subjected to determine the MICs against Gram-positive and Gram-negative selected bacteria. The results of the MIC experiment indicate that all compounds, and in particular the crude extract, had broad spectrum antibacterial activity (Figure 5), especially compounds pyranonigrin A (**1**) and fonsecin (**2**), which had the strongest inhibitory effect on *E. coli* at 21 ± 0.40 and 15 ± 0.15 μg/mL, respectively. Compound **1** pyranonigrin A had the lowest effect on the other types of the tested bacteria; *B. subtilis*, *S. aureus*, and *P. aeruginosa* had values of MIC ranging from 13 ± 0.35 to 152 ± 2.45 μg/mL. In addition, compound **4** asperazine had the best effect against *B. subtilis* (ATCC 6633), with an inhibitory effect of 8.5 ± 0.22 μg/mL. Overall, *P. aeruginosa* and *E. coli* bacteria were the most sensitive strains against tested compounds. MIC values of ampicillin as a positive control were determined at (1.1 ± 0.10, 3.5 ± 0.22, and 4.2 ± 0.35 μg/mL) in *S. aureus, B. subtilus,* and *E. coli,* respectively, whereas the positive control gentamicin had a measured MIC value of 8.5 ± 0.42 μg/mL in *P. aeruginosa*.

#### 2.6.2. Time–Killing Kinetics Assay

**Figure 6:**
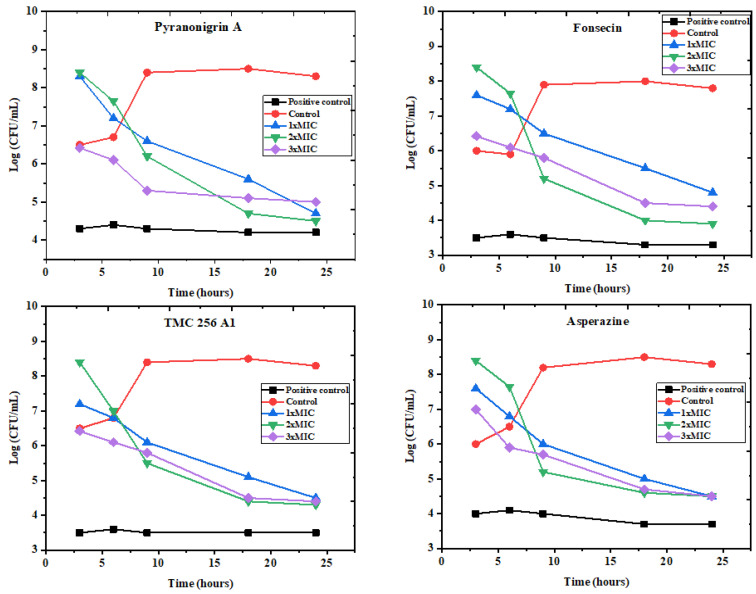
The time–kill kinetics of pure compounds (**1**–**4**) obtained from the active fraction of *A. tubingensis* (NA103) against *P. aeruginosa.* Red control indicates cells without treatment, while positive control indicates treated cells with 50 mg/mL of tetracycline.

**Figure 7:**
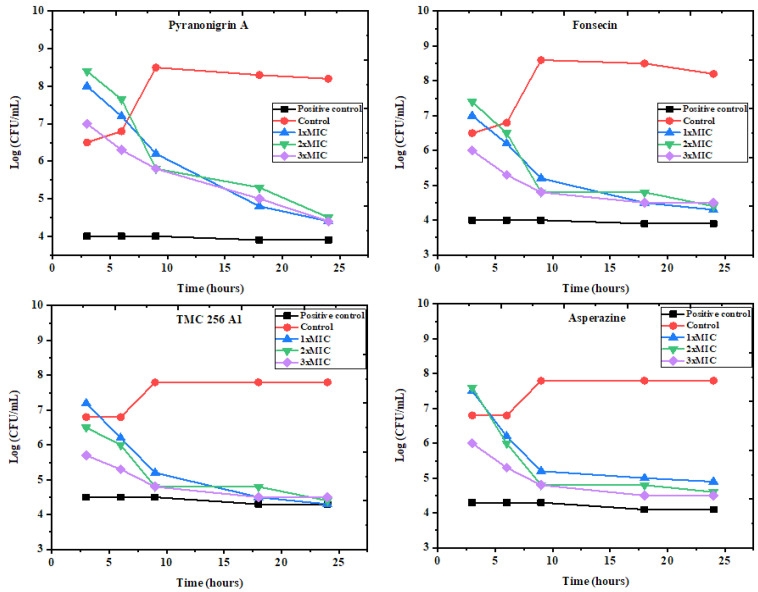
The time–kill kinetics of pure compounds (**1**–**4**) obtained from the active fraction of *A. tubingensis* (NA103) against *E. coli.* Red control indicates cells without treatment, while positive control indicates treated cells with 50 mg/mL of tetracycline.

The time–kill test is a highly appropriate method used for determining the antibacterial effect of tested compounds against various selected pathogens. This assay was studied over 24 h against the most sensitive bacteria, *P. aeruginosa* and *E. coli*, revealing the MIC values of investigated pure compounds **1**–**4** (112, 38, 63, 30, and 21, 15, 58, 32 μg/mL), 2 MICs (224, 76, 126, 60, and 42, 30, 116, 64 μg/mL), 3 MICs (336, 114, 189, 90, and 63, 45, 174, 96 μg/mL), respectively. As shown in (Figure 6 and Figure 7), the results are illustrated as the incubation periods and logarithmic number of CFU/mL. At all three MICs, all the pure compounds of the active fractions greatly exhibited *E. coli* at 3–6 h, which gradually rose up to 24 h compared with the MIC and two MICs and the control, respectively, while in *P. aeruginosa*, the two MICs and three MICs showed reasonable inhibition after 3 h, which gradually up to 24 h compared to the MIC and the control of most of the pure compounds except pyranonigrin A (**1**).

#### 2.6.3. Antifungal Activity

**Figure 8:**
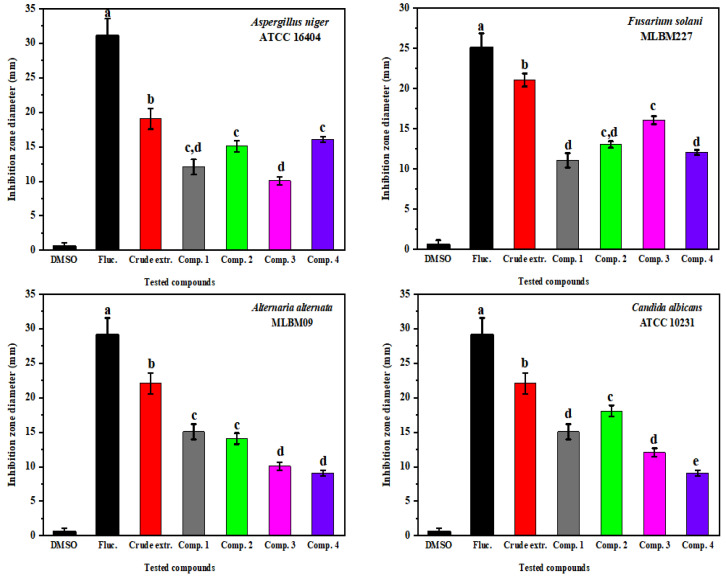
Antifungal activity of pure compounds and the crude extract against tested fungal pathogens. Error bars represent standard error of the means (*n* = 3).

The antifungal activities of the pure compounds and the fungal crude extract were also evaluated to determine the inhibition growth of the selected fungal pathogens. The results indicate that all compounds and in particular the crude extract had broad-spectrum antifungal activity (Figure 8), especially the crude extract, which had the highest inhibitory effect on all tested fungi with zones of inhibition ranging from 18.00 ± 0.85 to 22.50 ± 1.20 mm. Our findings demonstrate that tested fungi were sensitive to all the evaluated pure compounds, showing significant sensitivity with different degrees of inhibition. Compounds TMC 256 A1 (**3**) and asperazine (**4**) showed the maximum effect against *F. solani* MLBM227 and *A. niger* ATCC 16404 with inhibition zones of 16.40 ± 0.55 and 16.20 ± 0.20 mm, respectively, whereas the same compounds exhibited the lowest effect with inhibition zones of 12.30 ± 0.25, 10.0 ± 0.22 mm, and 9.0 ± 0.15, 9.3 ± 0.12 mm on *C. albicans* ATCC 10231 and *A. alternata* MLBM09, respectively. Moreover, compound fonsecin (**2**) had the best effect against *C. albicans* (ATCC 10231), with an inhibition zone of 17.8 ± 1.35 mm, and the compound pyranonigrin A (**1**) showed moderate activity on all tested fungi with zones of inhibition that ranged from 11.0 ± 0.60 to 15.2 ± 0.72 mm. The MIC values indicated that the purified compounds, in addition to the crude extract of NA103, exhibited varied MIC values ranging from 3.2 ± 0.15 to 14.8 ± 2.01 mg/mL against tested pathogenic fungal strains. The isolated compounds exhibited significant activity against *A. niger* ATCC 16404, whereas the lowest activity was observed against *C. albicans* ATCC 10231. The MIC results were calculated with the average of three replications and are listed in Table 1.

#### 2.6.4. Cytotoxicity Activity

**Figure 9:**
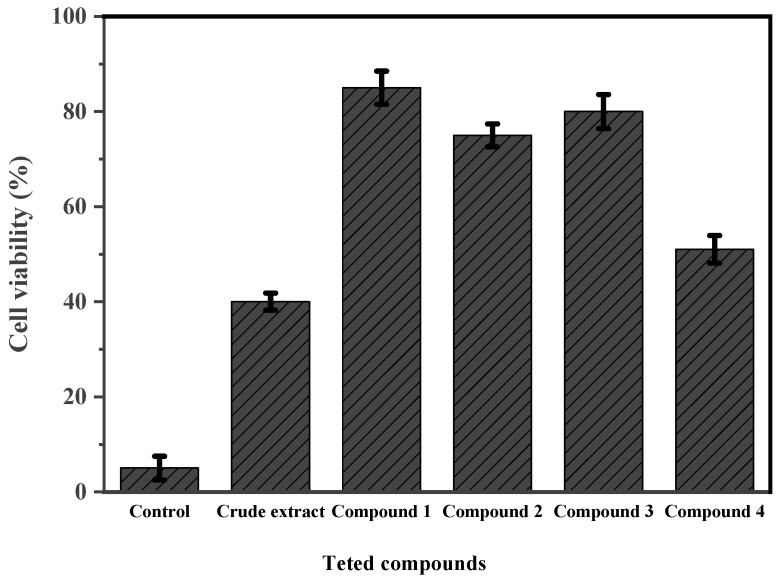
The MTT cytotoxic activity results for tested pure compounds and the crude extract of *A. tubingensis* (NA103) against L5178Y cell lines. Error bars represent standard error of the means (*n* = 3).

In the present experiment, the cytotoxic activity of the crude extract and pure compounds of the *A. tubingensis* (NA103) active fraction was evaluated using the MTT assay against mouse lymphoma cell line L5178Y. From the obtained results and in the presence of Kahalalide F as a positive control, it was found that, when L5178Y growth in % reached (10 µg/mL), the fungal crude extract and asperazine (**4**) exhibited moderate cytotoxicity with a cell viability percentage of (39 and 51%, respectively), while other tested pure compounds pyranonigrin A (**1**), fonsecin (**2**), and TMC 256 A1 (**3**) reported weak cytotoxicity (85, 74, 81%), respectively. The cytotoxicity activity results are illustrated in Figure 9.

## 3. Discussion

Endophytic fungi are an especially interesting group of microorganisms that can be obtained from plant tissues without any symptoms. Among them, most species belong to the ascomycetes and deuteromycetes classes and might be potential cell factories of multiple groups of novel and unusual secondary metabolites [22,23]. The fungal endophytes associated with their host plants as a specific relationship contain special chemical–biological interactions [24]. They have modified themselves to their particular environments via genetic variation after a long period of existence with their host plants and can synthesize biologically active molecules similar to the secondary metabolites produced by the host plants [25,26]. Multiple promising applications, mainly medicinal and agricultural, have been demonstrated using new biologically active substances obtained from several endophytic fungi. These compounds play a vital role in communication between organisms, both in plant adaptation to environmental changes and habitat and plant protection [24,27].

Many fungi that cause fruit spoilage have been identified after isolation from different niches. Similarly, *A. tubingensis* was sequestered from peach [28], and *A. niger* is a fungus frequently present on tomatoes [29], grapes [30], and apples [31]. Bali et al. reported that black mold *A. niger* was the main cause of post-harvest spoilage in acid lime and sweet orange in the field [32]. As determined by our observations during molecular identification of fungal isolates, *A. niger* and *A. tubingensis* were the most common black *Aspergillus* species. By virtue, our results confirm that this species was *A. tubingensis*, as it was similar to the described and identified strain K38 with regard to morphological and ITS region sequence homology [33]. Our findings are in good agreement with the results obtained by other authors; Peng et al. examined strain B, identifying it as *A. tubingensis* through morphological identification, and the homology was as high as 99% after identification by the method of molecular biology [34].

Black aspergilli are excellent candidates for a large variety of secondary metabolites, including NγPs, aflatoxins, fumonisins, cyclopiazonic acid, ophiobolins, and other important metabolites. A large number of these active compounds display interesting biological properties, such as antimicrobial, antioxidant, anticancer, anti-hyperuricuric, anti-HIV, and antitubercular properties [35,36]. In a recent study by El-hawary et al., they investigated the fermented culture of *A. tubingensis*, an endophytic fungus associated with *Lycium ruthenicum*, which yielded four known pyrone derivatives. Among them, rubrofusarin B had strong antibacterial activity against Gram-negative *E. coli* with an MIC value of 1.95 μg/mL, while other pure compounds showed weak antibacterial activities against *Streptococcus lactis*, *P. aeruginosa*, and *S. aureus*, in addition to *E. coli*, with MIC values ranging from 62.5 to 500 μg/mL [37]. Isolation and identification of pyranonigrin, a secondary metabolite, on the basis of spectroscopic studies on *A.* *niger* NBRC5374 and *A. niger* LL-LV3020 strains when grown on a solid culture, were also reported in previous studies [38,39]. Moreover, pyranonigrin A was isolated from an endophytic fungus *P. brocae* strain MA-231, associated with fresh tissue of the *Avicennia marina*, a marine mangrove plant, and showed significant antimicrobial activity against a broad spectrum of multiple human and plant pathogens [40].

The EtOAc crude extract of an endolichenic fungus, *A. tubingensis*, associated with *Parmelia caperata*, was evaluated against some clinically significant human pathogens; the results show significant antifungal activity against *Trichophyton mentagrophytes, C. albicans*, and *C. krusei* with an inhibition zone ranging from 25.30 ± 1.73 to 26.60 ± 0.57 mm, and moderate-to-strong antibacterial activity against *S. aureus*, *B. subtilis*, *P. aeruginosa*, *P. vulgaris*, and *K. pneumoniae* with a zone of inhibition ranging from 15.56 ± 0.32 to 20.30 ± 0.57 mm [41]. In another study, the TMC256A1 showed IL-4 signal transduction inhibition produced by *A. carbonarius*, *A. tubingensis*, and *A. niger* [42,43]. Moreover, Shaaban et al. demonstrated the biological activities of fonsecin obtained from *A. tubingensis*, *A. carbonarius*, *A. fonsecaeus*, *A. niger*, and *A. alternata* fungal strains, which possessed antimycobacterial [44]. Additionally, fonsecin has been isolated from *A. tubingensis* strain G131 and found to possess potent antioxidant activity against DPPH radicals, which agrees with the results reported by Leutou et al. [45]. Similarly, pyranonigrin A was reported to have antioxidant activity as a 1,1-diphenyl-2-picrylhydrazyl radical scavenging reagent [46]. Therefore, the antimicrobial metabolites from an endophytic fungus, *A. tubingensis* AN103, could be an important alternative biosource to overcome the increasing risk levels of drug resistance of significant human pathogens.

Our cytotoxicity assay showed moderate cytotoxicity of asperazine against the mouse lymphoma cell line L5178Y, while other pure compounds were reported to have weak cytotoxicity. Asperazine was isolated previously from *A. tubingensis* and was found to exert cytotoxic activity against leukemia cells but did not show reasonable antimicrobial activities [47]. The chemical analysis of extracts of 177 *A. tubingensis* strains, 140 *A. niger* strains, 47 *A. acidus* strains, and 1 *A. vadensis* strain reported production of asperazine only by *A. tubingensis* and *A. acidus*, which indicates the biosynthesis of asperazine can be considered a differential marker to distinguish multiple toxic black aspergilli from other low-toxicity strains, such as *A. tubingensis* and *A. acidus* [48]. In a recent study, naphthopyrones TMC-256A1 and fonsecin B displayed individually moderate in vitro cytotoxicity against the liver cancer HepG2 cell line, with values ranging between IC 50 and 30 μg/mL. In addition, these two compounds exhibited no antimicrobial activity against *S. aureus*, *B. subtilis*, *P. aeruginosa*, or *S. cerevisiae* [49]. Similar to our findings, the NGP fractionation of *A. tubingensis*, which showed moderate cytotoxicity, was mainly dependent on malformin A1 metabolite and not to any of the following NGPs present in the extract, including TMC 256A1, asperpyrone D, fonsecin B, rubrofusarin B, fonsecinone A, fonsecin, aurasperone E asperpyrone A, dianhydro-aurasperone C, and aurasperone A; these NGPs had no toxicity on various types of normal human fibroblast or cancer cell lines when tested with a concentration of 5 μg/mL on cell cultures [50]. Taken together, these properties demonstrate that obtained metabolites may be very useful as drug treatments and biological control agents for clinical bacterial and fungal pathogens, and the above investigation provided good evidence for our research on antimicrobial and cytotoxic activities in this article.

## 4. Materials and Methods

### 4.1. Site Description

Saratov Oblast (51°32′26″ N 46°00′30″ E) is a federal subject of the Russian Federation and is located in the Volga Federal District in the southeast part of European Russia. From west to the east, its territory stretches for 575 km, and from north to south for 330 km, with chernozem and chestnut soils. Five historic orchard sites were selected for sample collection: Saratov, Balashov, Bazarno-Karabulaksky, Engels District, and Pugachev. The selection of these sites was based on the opinions of local experts regarding the presumed history of these orchards as well as visually different morphologies of the apple trees.

### 4.2. Sampling and Isolation Procedures

A total of 40 randomly stem parts from fully matured apple trees (*M. domestica*) were collected from five different places in the Horticulture Research Farm of National Research Saratov State University and labeled upon completion of the ocular examination according to its location. They were placed in the plastic containers (30 cm diameter × 15 cm height), which were tightly covered. The containers then were kept at the laboratory temperature (26 ± 2 °C), and relative humidity was maintained at 60 ± 10%. To clean the dirt, samples were washed under running tap water and dried with sterile wipes. Briefly, stems were soaked in the solution of 70 % alcohol for 5 min, 0.5 % NaOCl for 3 min, and rinsed with sterile distilled water 3 times for 15 sec each time, and then dried using sterile wipes. A total of 100 mg of each stem was weighed out, ground, and mixed with 1 mL of saline solution using a mortar. From the mixture, 0.1 mL was plated on potato dextrose agar (PDA) (potato, 200 g/L, dextrose 20 g/L, agar 15 g/L, distilled water, 1000 mL), aseptically, and then incubated at 28 °C for 5–7 days. Morphological different and frequently appearing fungal colonies were randomly selected and purified. Each isolate was stored in PDA slants and kept at 4 °C for further study.

### 4.3. General Experimental Procedures

Mass spectra (ESI) were recorded with a Finnigan LCQ Deca mass spectrometer, and HRMS (ESI) spectra were obtained with FTHRMS-Orbitrap (Thermo-Finnigan, Weiler bei Bingen, Germany) mass spectrometer. All solvents that were used for separation processes and spectroscopic measurements were distilled and of spectral grade, respectively. For HPLC, analysis pump (Dionex UltiMate-3400 SD with LPG-3400SD) coupled to a photodiode array detector (DAD3000RS) was used, and detection was carried out at 235, 254, 280, and 340 nm. Eurosphere-100 C18 (Knauer, Berlin, Germany) prefilled column (125 mm—4 mm) was used for separation. The following gradient was used (MeOH, 0.02% H_3_PO_4_ in H_2_O): 0 min. (10% MeOH), 5 min. (10% MeOH), 35 min. 100% Methanol (MeOH), and 45 min. (100% MeOH). For purification, Hitachi HPLC System (UV detector L-7400; Pump L-7100; Eurosphere-100 C18, 300 mm, 8 mm, Knauer, Berlin, Germany) was used as semi-preparative HPLC to obtain the final purifications. Column chromatography included LH-20 Sephadex and Merck MN Silica gel 60 M (0.04–0.063 mm), and thin-layer chromatography (TLC) was carried out using normal phase silica gel F254 (Merck, Darmstadt, Germany) plates, and detection was performed under UV at 254 and 366 nm, confirmed by spraying the plates with anisaldehyde reagent.

### 4.4. Screening for Fungal Isolates and Their Biological Activity

After isolation procedures, all purified fungal isolates growing on agar blocks (3 mm in diameter) were sub-cultured in 250 mL Erlenmeyer flasks that contained 100 mL of potato dextrose yeast extract broth medium and incubated in a rotary shaker for 7 days at 28 °C with agitation speed at 150 rpm. After cultivation period, fungal mycelia were separated and filtered using a Whatman filter (0.45 μm) and extracted by liquid–liquid extraction with an equal volume of ethyl acetate (EtOAc) extract. The ethyl acetate layer was further concentrated by evaporating to dryness at 40–45 °C, and (100 mg/mL) the resultant residue was dissolved in 1 mL of 10% dimethyl sulphoxide (DMSO) for antibacterial assay. All fungal extracts were screened for qualitative antibacterial determination against different pathogenic microbes by the disk diffusion method as previously described [51]. Briefly, each dissolved extract was sterilized and filtered using a syringe filter (pore size of 0.22 μm), and sterile blank discs (Whatman No. 1, diameter 6 mm) were saturated with (10 mg/mL) of extract, and the impregnated discs were then kept at 4 °C before use. Extract-saturated discs were applied on Mueller–Hinton agar plates and incubated for 24 h at 37 °C. Tetracycline discs (50 µg/mL) and 10% DMSO-saturated discs were used as positive and negative control, respectively. After incubation, the plates were observed for bio-activity against tested bacteria; the most promising strain demonstrating the highest activity (zone of inhibition) was selected for further analyses.

### 4.5. Identification of Endophytic Fungi

#### 4.5.1. Morphological Analysis

For morphological examination, all obtained fungi were streaked on plates of PDA medium and incubated for 5–7 days at 28 °C. Microscopic observations were carried out to check the structure and purity of the fungal isolates with the help of lactophenol cotton blue-stained mycelia. The morphological characteristics were observed based on their colony morphology and growth features with reference to fungi to identify the species levels [52,53]. The promising strain (with the highest bioactivity) was selected for further molecular identification.

#### 4.5.2. Molecular Identification of the Selected Strain

Genomic DNA was isolated from the selected fungus using the DNA quick Plant System isolation kit (Tiangen, Biotech, Beijing, Co., Ltd., Beijing, China). ITS1 (TCCGTAGGTGAACCTGCGG) and ITS4 (TCCTCCGCTTATTGATATGC) primers were used, and the specific ITS region of the target isolate was amplified by PCR [54]. The 2X Accurate Taq Master Mix Kit (Qiagen, Shanghai, China) was performed for PCR amplification. PCR reaction mixture was performed in a total volume of 50 μL containing PCR Master Mix (25 μL), template DNA (2 μL), taq polymerase (1 μL), and 1 μL each of ITS1 and ITS4, with the final volume of the reaction mixture being adjusted with DNase-free water (20 μL). PCR condition was as follows: DNA denaturation at 95 °C for 5 min, followed by 35 cycles at 98 °C for 15 s, annealing at 56 °C for 30 s, and extension at 72 °C for 1 min, and a final extension at 72 °C for 5 min. The PCR products were separated on agarose TBE-gels (Tris-base Boric EDTA-gel) and purified by a QIA quick DNA gel extraction kit (Omega Bio-tek, Guangzhou, China) according to the manufacturer’s instructions. Sangon Biotech (Shanghai, Co., Ltd., Shanghai, China) sequenced the amplified PCR product, and the resulting sequences were submitted to the NCBI database. The phylogenetic analyses inferred from the data were constructed using MEGA-X software version 11.0.6. [55].

### 4.6. Scanning Electron Microscope (SEM)

The fungal isolate was cultured on PDA medium at 28 °C for 5 days. The fungal mycelium was picked up on a 200-mesh copper screen and dried for viewing in the scanning electron microscope. SEM measurements were carried out using ion sputtering device MIRA II LMU instrument, and observed at 15–20 KV, using an XL30 ESEM FEG (Philips, New York, NY, USA) device at 15 KV. Micromorphology was studied by taking images at magnification varying from 100- to 40,000-fold. This experiment was performed at the electron microscope unit of National Research Saratov State University, Russian Federation.

### 4.7. Cultivation, Extraction, and Isolation of Secondary Metabolites

The selected strain was cultivated on PDA at 28 °C for 5–7 days. Fermentation was carried out in solid rice medium (100 g of rice and 110 mL of H_2_O in 1 L Erlenmeyer flasks), and autoclaved at 121 °C for 20 min. After cooling down to room temperature, small pieces of agar that contained fungal spores were transferred to flasks and incubated at 25 °C under static conditions for 30 days. The fermented rice medium was extracted by adding (500 mL × 3) EtOAc to each flask. EtOAc extracts were filtered by using a vacuum and washed with nano-pure water and then evaporated till dry (at 40 °C in rotary evaporator). Then, the crude extract was fractioned between 90% methanol (MeOH) and *n*-hexane (C₆H₁₄), and 90% methanol fraction was dried to give (2.5 g) solid residue. The solid residue was dissolved in MeOH (10 mL), and filtered by 0.45 μm microporous membrane, and the filtrate-containing compounds were loaded on a Sephadex LH-20 column eluted with 100% MeOH, yielding four fractions (Figure 1, Figure 2, Figure 3 and Figure 4). Based on fractions’ preliminary antibacterial activity against multiple human pathogenic bacteria, fraction no. 2 (Figure 2) was determined to be the most active fraction and further subjected to semi-preparative RP-HPLC and eluted with MeOH:H_2_O in (70–100%, *v*/*v*) gradient to afford four different compounds: **1**, **2**, **3,** and **4**.

### 4.8. Determination of Biological Assay

#### 4.8.1. Antibacterial Activity

Test organisms used to evaluate the antibacterial effects of isolated compounds were obtained from the American Type Culture Collection (ATCC). The selected human pathogenic bacteria were *B. subtilis* ATCC 6633, *S. aureus* ATCC 6538P, *E. coli* ATCC 8739, and *P. aeruginosa* ATCC 9027. The antibacterial activity testing was carried out using the 96-well microplate assay as previously described [56]. The bacterial species were grown in Mueller–Hinton broth (MHB) medium (2.0 g/L beef infusion solids; 1.5 g/L starch; 17.5 g/L casein hydrolysate; distilled water 1000 mL; and final pH 7.4 ± 0.2) under aerobic conditions by shaking at 200 rpm and incubated overnight at 37 °C. All tested compounds as well as crude extract were dissolved in 10% DMSO and diluted into a stepwise concentration (50, 40, 30. 20, and 10 μg/mL). The 96-well microplate was measured by an absorbance plate reader (UV/Vis Multiskan Sky Microplate Spectrophotometer, ThermoFisher Scientific^TM,^ Pudong New Area, Shanghai, China), after 12 h of incubation at 37 °C. Ampicillin and/or gentamicin (0.02 g/mL) was used as a positive control, and diluted pure DMSO was used as a negative control. MIC values were determined by testing all pure compounds in triplicate and defined as the lowest concentrations without visible bacterial growth.

#### 4.8.2. Time–Kill Kinetics

Time–kill assay was carried out using the MIC values of the most susceptible tested bacteria; the suspension of chosen strains was adjusted to bacterial serial ten-fold dilutions ranging from 10^3^ to 10^4^ (1.5 × 10^7^ cfu/mL), and antimicrobial activity was tested with concentrations of MIC values of 1 MIC, 2 MIC, and 3 MIC of the isolated pure compounds. Tetracycline (50 µg/mL) served as a positive control. Each treatment (80 µL) was put into a well containing 100 µL of MHB including 20 µL of bacterial suspension and incubated for 24 h at 37 °C. Samples were collected at five time points: 3, 6, 9, 18, and 24 h. Then, 100 µL of collected samples was sub-cultured on MHA and incubated for 24 h at 37 °C. Several viable bacterial cells were determined.

#### 4.8.3. Antifungal Activity

The antifungal assay of the crude extract and pure compounds was evaluated by the disc diffusion method. Blank sterile paper discs measuring 6 mm were impregnated with 20 μL of test concentration of the fungal crude extract, and isolated pure compounds and air-dried discs were transferred aseptically to respective plates. The activity was determined by the area of inhibition in millimeters (mm). The reference standard for antifungal activity was fluconazole discs (25 μg), while discs dipped in DMSO were used as a negative control. The antifungal activities against the pathogenic fungi, namely, *A. niger* ATCC 16404, *F. solani* MLBM227, *A. alternata* MLBM09 and *C. albicans* ATCC 10231, were evaluated. The choice of test strains was based on fungi’s opportunistic pathogenicity and resistance to conventional drugs [57]. MIC values were determined by testing all pure compounds in addition to crude extract in triplicate using sabouraud dextrose agar medium containing glucose (40 g/L), peptone (10 g/L), and agar (20 g/L) in 1000 mL of distilled H_2_O with final pH (5:8 ± 0:2) for *C. albicans* ATCC 10231, while PDA medium was used for other tested fungal strains. MIC experiments were carried out by the method described previously [58], and defined as the lowest concentrations without visible fungal growth.

#### 4.8.4. Cytotoxic Activity (MTT Assay)

The cytotoxicity of the obtained pure compounds besides the fungal crude extract was tested against L5178Y mouse lymphoma cells using the microculture tetrazolium (MTT) assay. Kahalalide F was used as a positive control, whereas DMSO was investigated as a negative control [59]. The cytotoxicity experiments were repeated and carried out in triplicate.

### 4.9. Statistical Analysis

All the experiments were conducted in triplicate, and one-way analysis of the variance was performed followed by Student’s *t* test. Results are presented as mean ± SD. All the calculations were carried out using OriginPro, program version 95 E, and *p* < 0.05 was used in statistical analysis to establish significant differences wheresoever applicable.

## 5. Conclusions

In this work, we investigated the bioactive metabolites from *A. tubingensis* associated with *M. domestica* trees. Different chromatographic techniques were used for isolation, separation, and identification of pure metabolites (**1**–**4**) from the fermented culture. These compounds were identified as pyranonigrin A, fonsecin, TMC 256 A1, and asperazine based on spectroscopic data, including 1D-NMR, 2D-NMR, and HR-ESIMS. These compounds had weak-to-moderate cytotoxic activity against lymphoma cell lines (L5178Y) and a significant antimicrobial activity against bacterial and fungal human pathogens. These results can be applied to the large-scale production and biotechnological application of potential drugs.

## Figures and Tables

**Table 1:** MIC values of fungal crude extract and its isolated compounds of *A. tubingensis* (NA103) against selected pathogenic fungi.

Tested Compounds	MIC Values (μg/mL)			
	*A. niger* ATCC 16404	*F. solani* MLBM227	*A. alternata* MLBM09	*C. albicans ATCC 10231*
**Crude extract**	4.3 ± 0.01	4.1 ± 0.03	3.2 ± 0.15	6.5 ± 0.28
**Pyranonigrin A (1)**	6.4 ± 0.22	5.5 ± 0.04	5.9 ± 0.17	12.4 ± 1.32
**Fonsecin (2)**	5.1 ± 0.25	6.1 ± 0.12	6.2 ± 0.20	9.6 ± 0.52
**TMC 256 A1 (3)**	4.9 ± 0.31	6.6 ± 0.22	8.1 ± 0.29	13.2 ± 1.62
**Asperazine (4)**	4.3 ± 0.02	4.8 ± 0.10	10.5 ± 0.45	14.8 ± 2.01

## Data Availability

Not applicable.

## Supplementary Materials

The following supporting information can be downloaded at: https://www.mdpi.com/article/10.3390/molecules27123762/s1. Table S1: Antibacterial activity of the ethyl acetate crude extracts of endophytic fungi against different pathogenic bacteria; Figure S1: The UV spectrum (A), ESI-MS positive mode (B), ESI-MS negative mode (C) of Pyranonigrin A (**1**); Figure S2: ^1^H NMR spectrum of Pyranonigrin A (**1**) measured in DMSO; Figure S3: The UV spectrum (A), ESI-MS positive mode (B), ESI-MS negative mode (C) of fonsecin (**2**); Figure S4: ^1^H NMR spectrum of fonsecin (**2**) measured in CDCl_3_; Figure S5: The UV spectrum (A), ESI-MS positive mode (B), ESI-MS negative mode (C) of TMC 256 A1 (**3**); Figure S6: ^1^H NMR spectrum of TMC 256 A1(**3**) measured in DMSO; Figure S7: The UV spectrum (A), ESI-MS positive mode (B), ESI-MS negative mode (C) of asperazine (**4**); Figure S8: ^1^H NMR spectrum of asperazine (**4**) measured in DMSO.
