# Supplementary material for: In Vitro Phytobiological Investigation of Bioactive Secondary Metabolites from the Malus domestica-Derived Endophytic Fungus Aspergillus tubingensis Strain AN103
Source: Molecules. 2022 Jun 11;27(12):3762. doi: 10.3390/molecules27123762 (PMC9228098; doi:10.3390/molecules27123762)
Supplement: Supplementary file 1 [file molecules-27-03762-s001.zip › molecules-1745234-supplementary.pdf]

Supplementary materials

Table S1. Antibacterial activity of the ethyl acetate crude extracts of endophytic fungi against different pathogenic bacteria

No.	Fungal isolates	Diameters of inhibition zone (mm)			
		E. coli	*P. aeruginosa*	*S. aureus*	*B. subtilis*
1	*Alternaria tenuissima* AT50	23.5±0.4	22.5±0.3	25.2±0.5	27.3±0.5
2	*A. tenuissima* AT57	24.2±0.3	24±0.2	25.8±0.6	26.5±0.4
3	*A. alternata* AA52	-ve	17.4±0.2	28±0.7	23.6±0.3
4	*A. alternata* AA55	16.4±0.4	16.5±0.3	27.5±0.6	22.6±0.2
5	*A. alternata* AA60	-ve	15.8±0.2	27.9±0.8	23.2±0.2
6	*A. brassicae* AB85	14.2±0.2	20.5±0.4	26.3±0.5	22.4±0.3
7	*A. solani* AS89	18.5±0.5	21.8±0.4	26±0.6	21.2±0.2
8	*A. mali* AM95	-ve	15.6±0.3	25.4±0.5	22±0.2
9	*Aspergillus niger* AN101	17.5±0.5	26.2±0.8	25.2±0.4	27.2±0.5
10	*A. niger* AN102	18±0.7	24.5±0.5	24.6±0.6	26±0.6
11	*A. tubingensis* AN103	25.7±0.7	27.4±0.6	28.4±0.7	29.5±0.8
12	*A. fumigatus* AF107	24.5±0.4	-ve	22.4±0.3	28.1±0.6
13	*A. terreus* AT105	22.4±0.5	22.7±0.3	23.6±0.4	26.2±0.5
14	*A. flavus* AF127	25.2±0.6	23.8±0.2	25±0.6	28.5±0.8
15	*Penicillium citrinum* PC131	27±0.7	19.6±0.2	23.4±0.4	27.5±0.5
16	*P. chrysogenum* PC133	25.1±0.2	17.2±0.3	26.3±0.4	26.2±0.4
17	*P. chrysogenum* PC134	25.2±0.4	22.8±0.5	22.4±0.3	25.5±0.4
18	*P. funiculosum* PF140	-ve	-ve	18.6±0.2	20.5±0.2
19	*Fusarium equiseti* FE145	23.4±0.3	18±0.2	21.9±0.2	27.4±0.4
20	*F. tricinctum* FT148	21.2±0.2	17.5±0.2	23.6±0.3	28±0.5
21	*F. oxysporum* FO150	22.5±0.2	22±0.5	22.8±0.3	19±0.4
22	*F. solai* FS162	20±0.2	-ve	20.4±0.4	16.5±0.3
23	*Cladosporium cladosporioides* CC168	-ve	17.5±0.3	21.2±0.5	22.7±0.7
24	*C. cladosporioides* CC169	-ve	16.1±0.2	20.5±0.4	23±0.6
25	*Cladosporium* spp. CC170	13.3 ±0.2	-ve	16.5±0.3	19.7±0.4
26	Positive control “Tetracycline ”	26.3	16.4	28.8	30.7

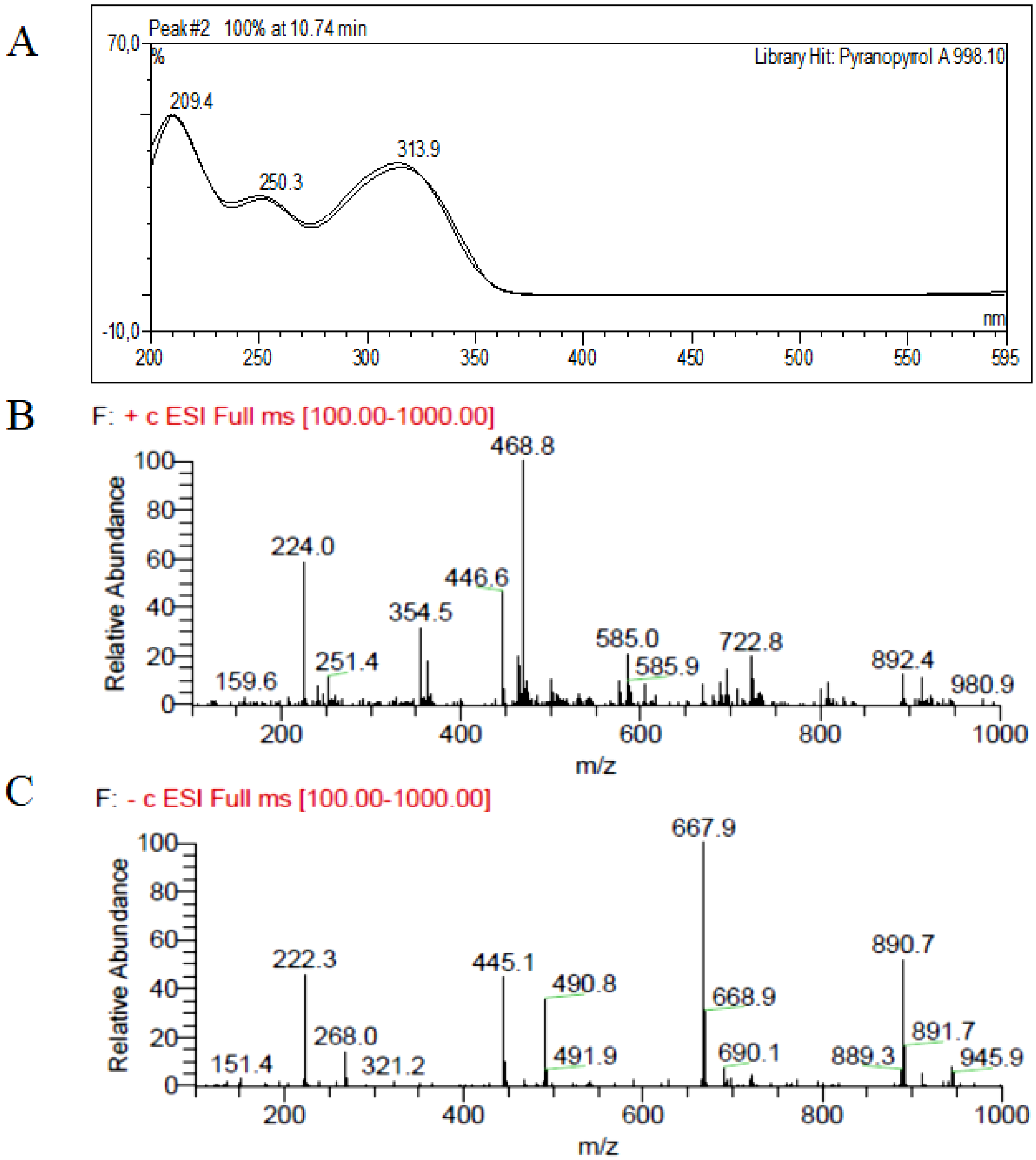

Figure S1. The UV spectrum (A), ESI-MS positive mode (B), ESI-MS negative mode (C) of Pyranonigrin A (**1**).

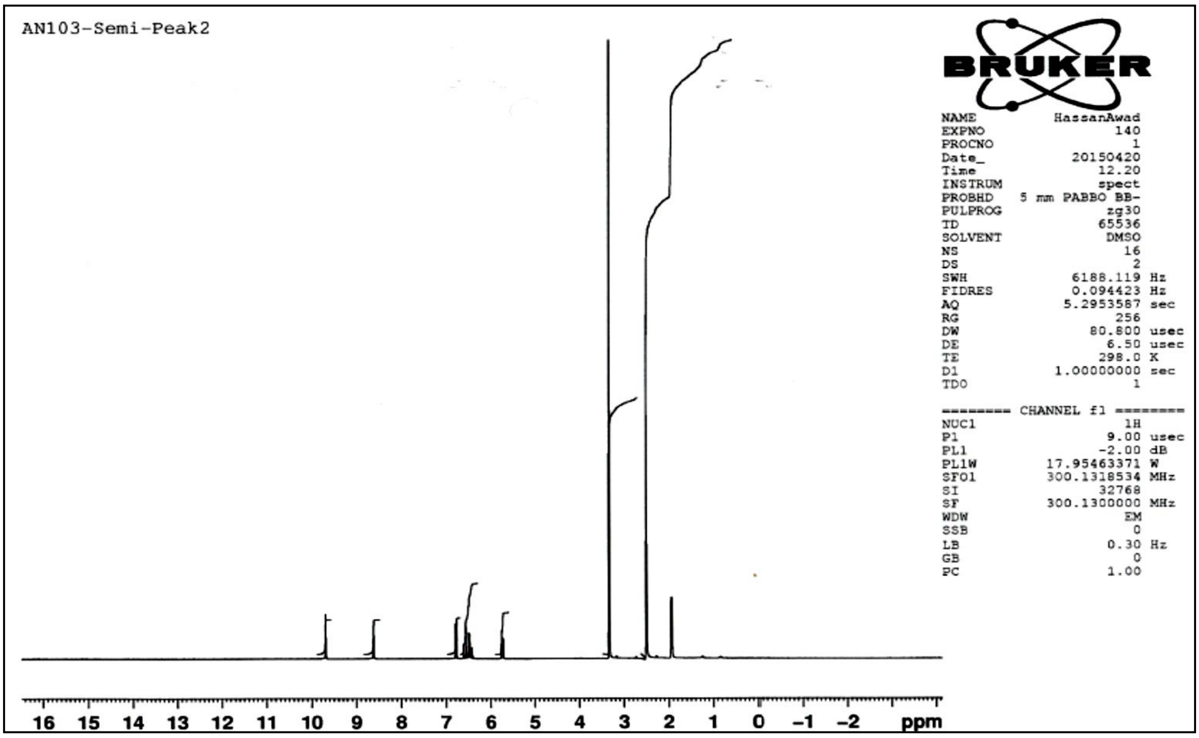

Figure S2. ^{1}H NMR spectrum of Pyranonigrin A (**1**) measured in DMSO.

A

B

C

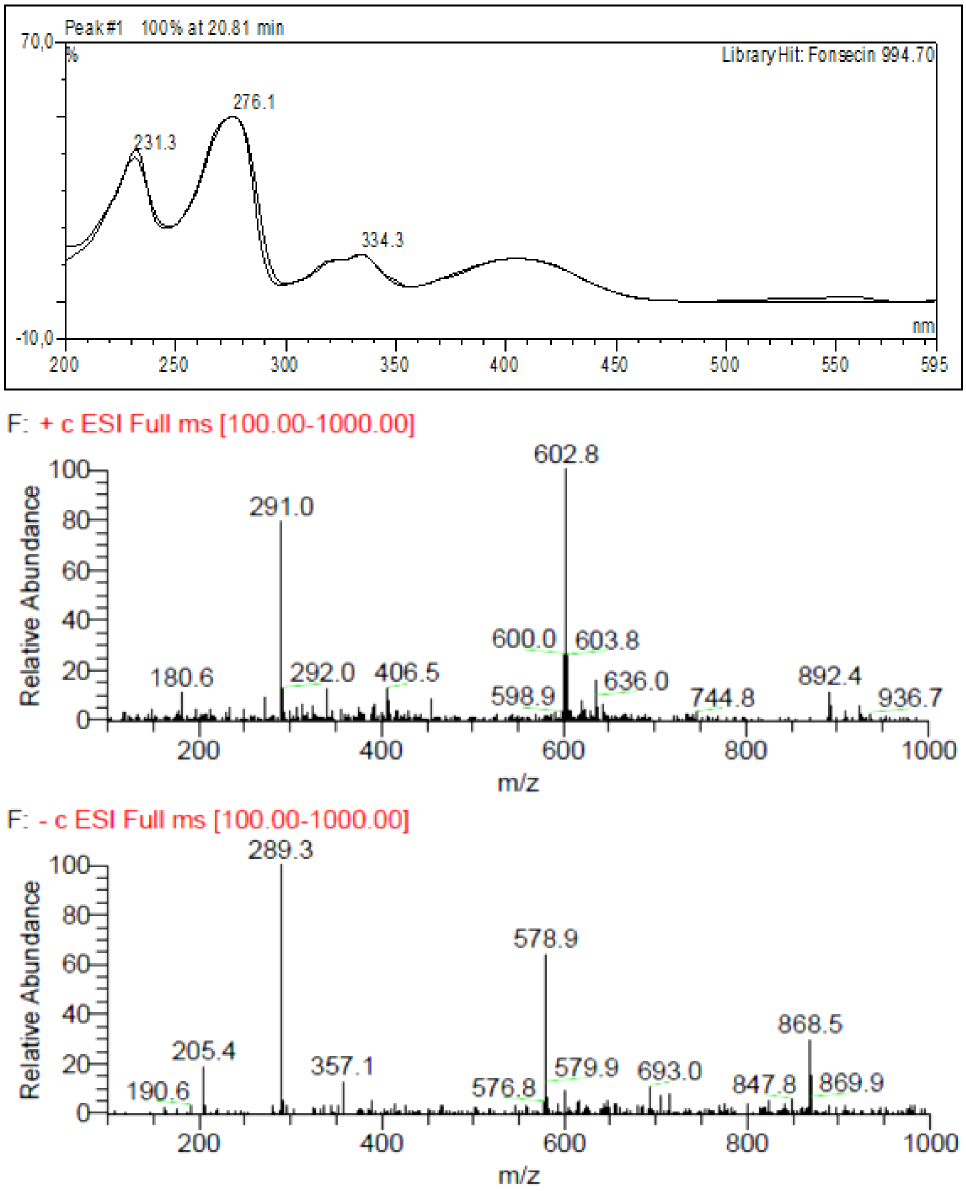

Figure S3. The UV spectrum (A), ESI-MS positive mode (B), ESI-MS negative mode (C) of Fonsecin (**2**).

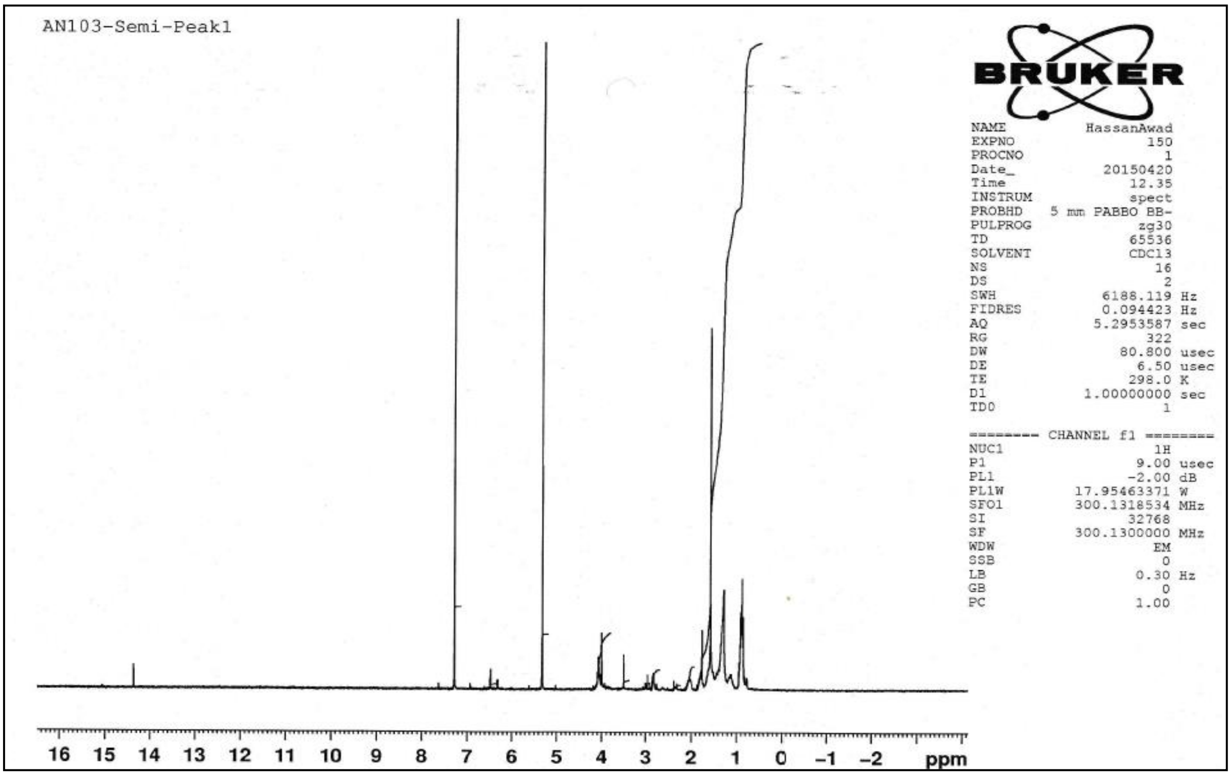

Figure S4. ^{1}H NMR spectrum of Fonsecin (**2**) measured in $CDCl_3$.

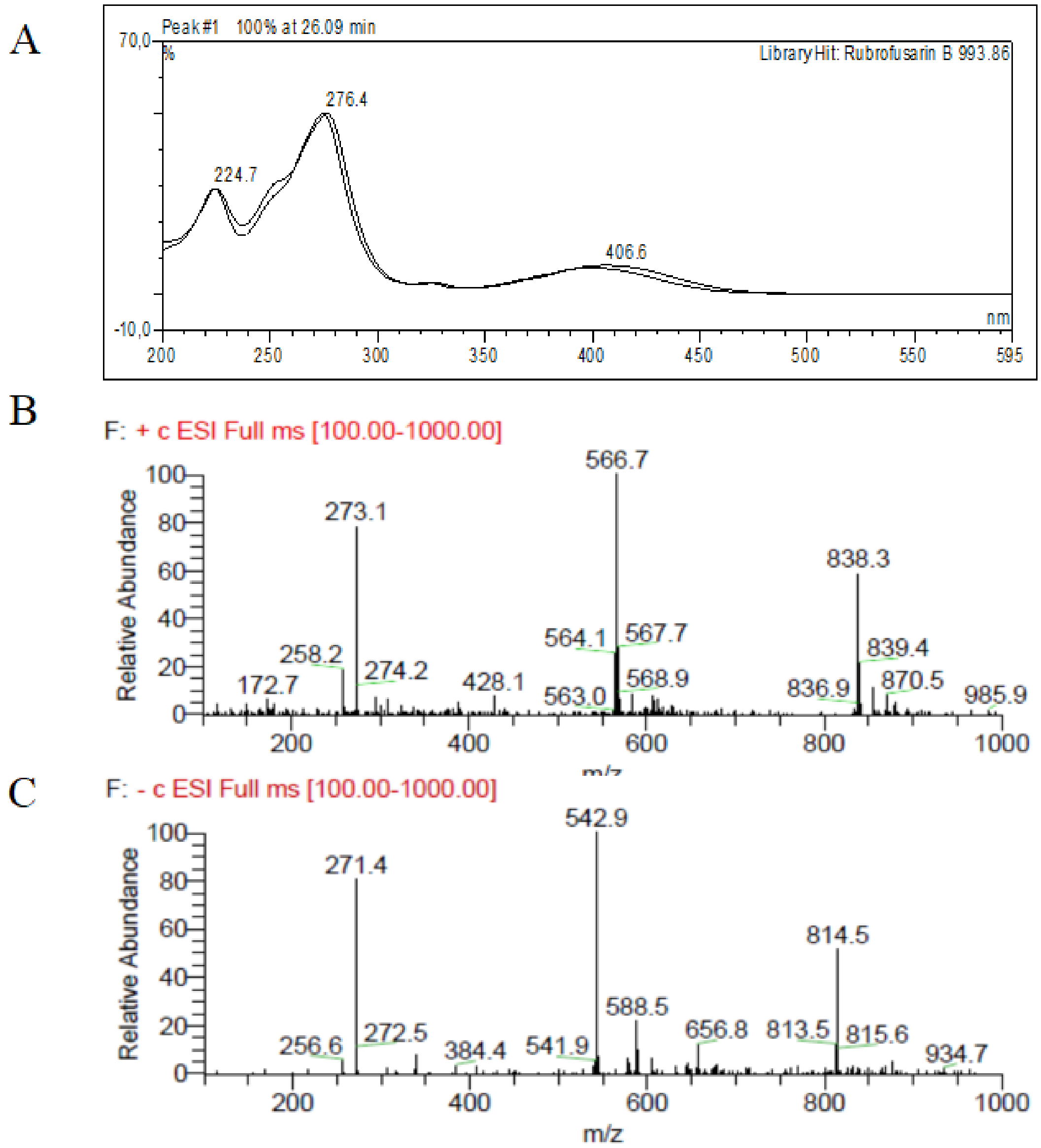

Figure S5. The UV spectrum (A), ESI-MS positive mode (B), ESI-MS negative mode (C) of TMC 256 A1 (**3**).

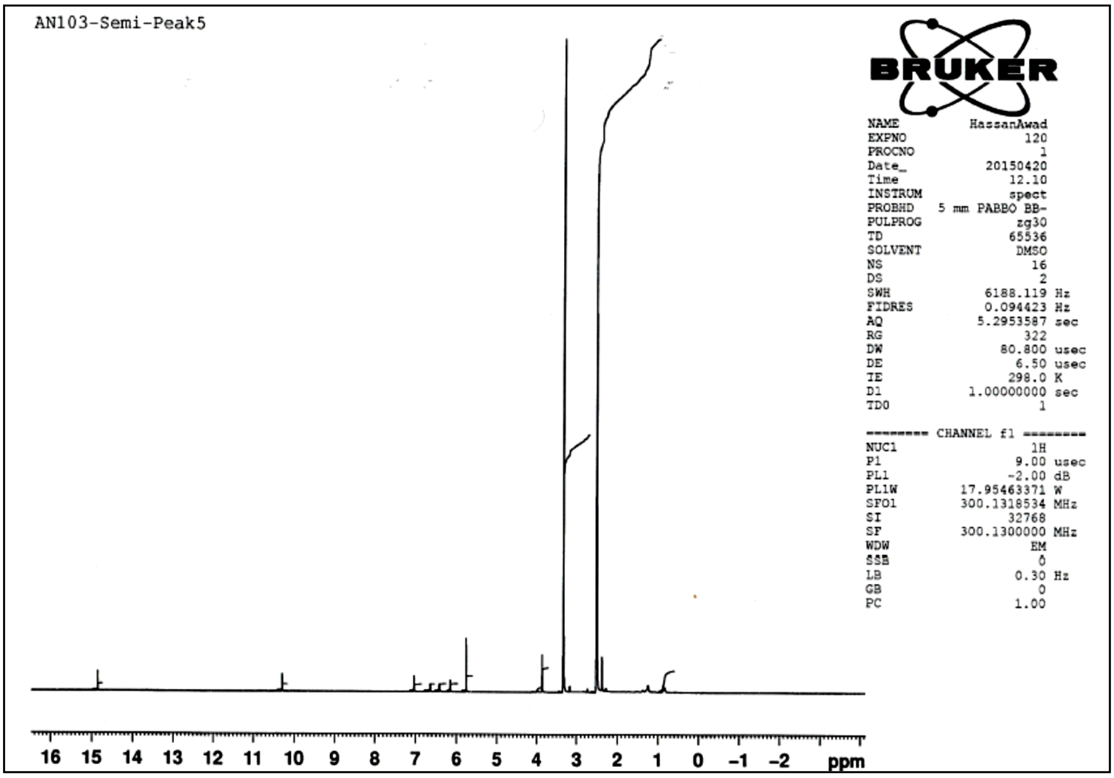

Figure S6. ^{1}H NMR spectrum of TMC 256 A1 (**3**) measured in DMSO.

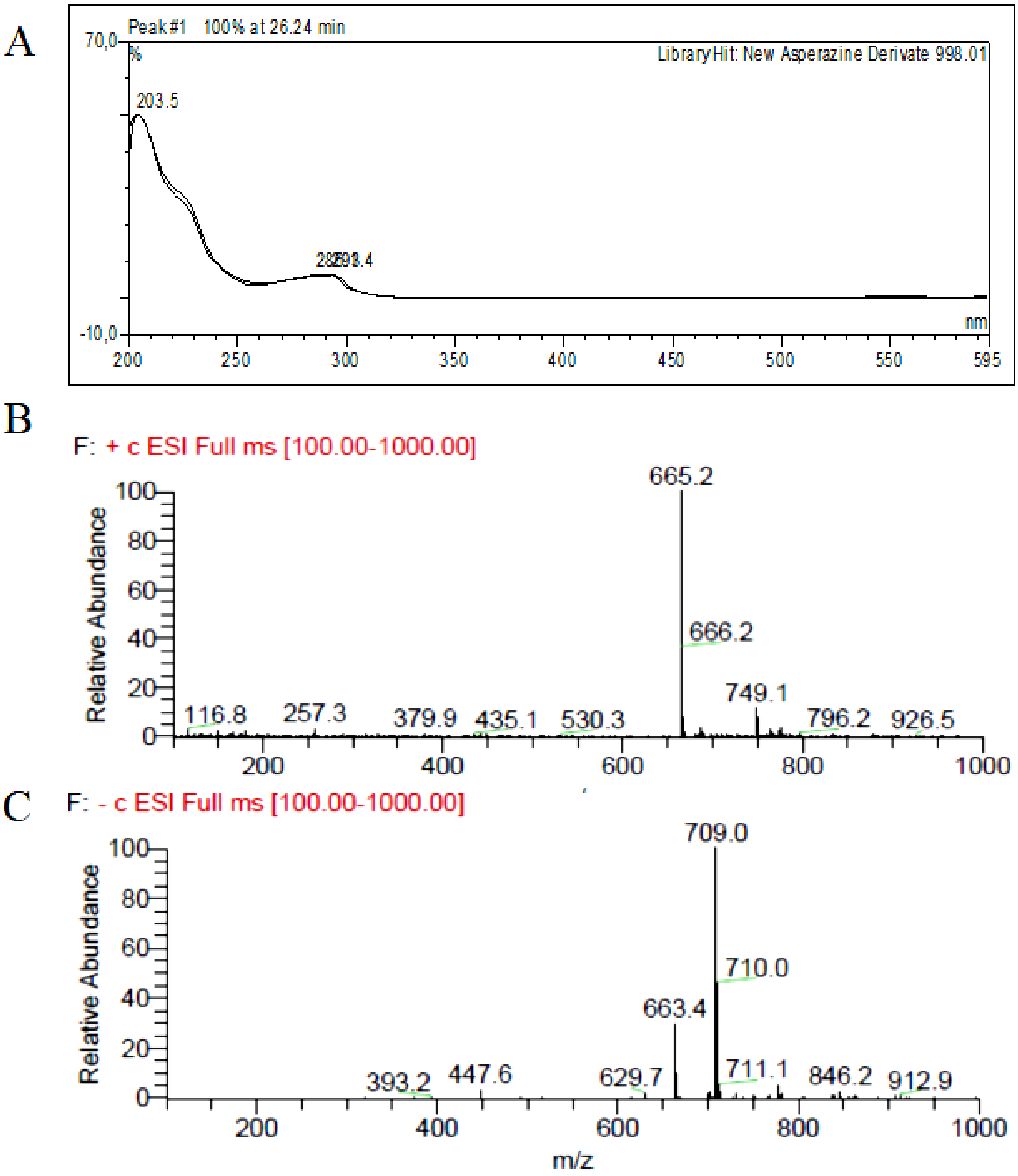

Figure S7. The UV spectrum (A), ESI-MS positive mode (B), ESI-MS negative mode (C) of Asperazine (**4**).

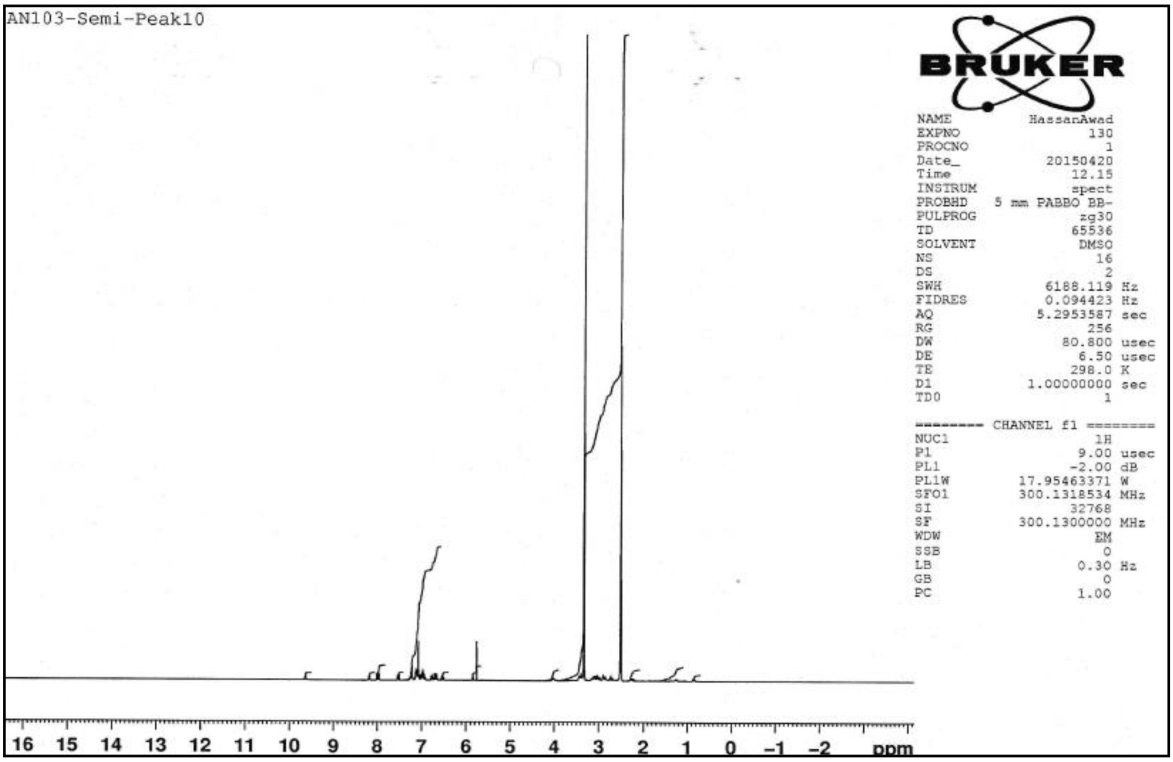

Figure S8. ^{1}H NMR spectrum of Asperazine (**4**) measured in DMSO.
